# Greater Glycaemic Response to an Oral Glucose Load in Healthy, Lean, Active and Young Chinese Adults Compared to Matched Caucasians

**DOI:** 10.3390/nu10040487

**Published:** 2018-04-14

**Authors:** Trevor Simper, Caroline Dalton, David Broom, Waleed Ibrahim, Lingjin Li, Charles Bankole, Sisi Chen

**Affiliations:** 1 Food and Nutrition Group, Sheffield Business School, Sheffield Hallam University, Sheffield S1 1WB, UK; waleedcdc@gmail.com (W.I.); li_lingjin@163.com (L.L.); Charlybanky@gmail.com (C.B.); anna911221@hotmail.com (S.C.); 2 Biomolecular Sciences Research Centre, Sheffield Hallam University, Sheffield S1 1WB UK; c.f.dalton@shu.ac.uk; 3 Academy of Sport and Physical Activity, Sheffield Hallam University, Sheffield S1 1WB, UK; d.r.broom@shu.ac.uk

**Keywords:** glycaemic response, iAUC, diabetes, ethnicity

## Abstract

There are ethnic differences recorded in glycaemic response and rates of type 2 diabetes mellitus (DM) between Chinese and Caucasian populations. Whether these differences are evident in matched healthy, lean, active, young adults is unclear. This study compares the postprandial glycaemic response of a group of Chinese participants (*n* = 49) with a group of similar Caucasians, (*n* = 48) aged 23.8 (±4.35 years), body mass index (BMI) 22.7 (±2.6) kg/m^2^, healthy (free from non-communicable disease), and lean (body fat % 23.28% (±5.04)). Participants undertook an oral glucose tolerance test to identify any significant differences in postprandial blood glucose response. Body fat percentage, body mass, age, physical activity, baseline glucose and HbA1c did not significantly differ between groups. Data from food frequency questionnaires indicated that the Chinese participants consumed less starchy foods, candy and “other” sweets and sugary drinks, and more rice than the Caucasians (all *p* ≤ 0.001), but not a greater overall intake of carbohydrates or any other macronutrient (all *p* > 0.05). The two groups’ postprandial blood glucose responses and 2-h incremental area under the curve values (iAUC)—156.67 (74.12) mmol/L 120 min for Caucasians versus 214.03 (77.49) mmol/L 120 min for Chinese—indicate significant differences (*p* = 0.003 and *p* < 0.001 respectively) between groups. Findings suggest that the difference between the two groups’ iAUC values do not relate to obvious lifestyle factors. The Chinese group were eating the least sugary and starchy food but had the highest iAUC. It is argued that the Chinese group in this investigation have the most favourable BMI, body fat percentage, and body mass, yet “poorest” glycaemic response.

## 1. Introduction

Diabetes prevalence in the UK rose from 1.4 million to 3.2 million people (type 1 and type 2 combined) between 1996 and 2013, with the majority being type 2 diabetes mellitus (T2DM); 90–95% of cases) [1]. Prevalence in China is higher than Europe, with estimates between 92.4 and 114 million adult cases which equates to 10–12% of the Chinese population [2,3]. The prevalence of pre-diabetes (impaired glucose tolerance) is more alarming, and according to Xu et al. [2] is 493.4 million people, equalling approximately 50% of the adult Chinese population.

Rapid changes to lifestyle and accelerated urbanization and industrialization have been highlighted as factors correlating with a vastly increased non-communicable disease pattern in China [4]. Key examples of the changes to Chinese society include a declining rate of both leisure time and occupational physical activity amongst adults [4] and poorer dietary intake (examples include increased intake of refined grains, reduced intake of coarse grains, increased fat and meat consumption, and a decline in vegetable intake) [5].

Elevated post-prandial glucose responses do not relate solely to populations with diabetes; increased risk of mortality also appears to be related to high glucose levels in pre-diabetic populations [6]. Despite evidence alluding to the significance of elevated post-prandial glucose levels in healthy individuals, there has been greater focus on examining individuals with diabetes. Therefore, blood glucose responses in seemingly healthy Chinese and Caucasian individuals needs comparison and consideration.

The potential difference between blood glucose responses in Chinese and Caucasian populations has been highlighted previously [7,8]. Chan and colleagues [9] assessed the glycaemic index of foods, comparing Caucasian and Vietnamese participants’ responses, and concluded that glycaemic index (GI) values derived from Caucasian participants are likely to be translated to people from Asian populations. Tan and colleagues also looked at ethnic differences in glycaemic response between Malay, Chinese, and Asian-Indian participants, and found similar glucose responses in all three groups when using different varieties of rice as a test food [10]. Yip et al. reviewed work carried out on impaired fasting glucose (IFG) and impaired glucose tolerance (IGT) across ethnicities, concluding that the prevalence of IFG was higher in Caucasian cohorts and IGT, and combined IFG and IGT were higher in Asian Chinese cohorts [11]. Impaired glucose tolerance relates to having elevated blood glucose 2 h after ingestion of a standard dose of glucose (often 50 or 75 g); a cutoff point of 7.8 mmoL/L following a 50 g dose of glucose and 8.0 mmol/L for 75 g has been previously observed [12]. Impaired fasting glucose relates to the level of blood glucose being between 6.1 and 6.9 mmol/L when the subject has fasted overnight [12].

Other investigations which have compared the glycaemic response—as opposed to glycaemic index—to different foods or glucose suggest an ethnic difference in responses resulting from carbohydrate ingestion [13]. Henry et al. [14] found a clear difference in the incremental area under the curve (iAUC) between Asian-Indians and European participants, but no difference in the GI of the foods consumed. Essentially, the glycaemic index appeared to be transferable between ethnicities (i.e., the percentage rise in blood glucose from test foods relative to glucose was similar between ethnicities), but the overall increase in blood glucose was significantly higher in the Asian-Indian participants. Kataoka et al. [8] focused specifically on rice and rice variety, concluding that the glycaemic responses were appreciably higher in Chinese compared to Caucasian participants, although the Caucasian participants were significantly more physically active and higher in body mass index (BMI)/body mass in the Kataoka study and it is uncertain to what degree the weight difference was related to adipose or muscular tissue. The present investigation adds to this by matching participants for obvious factors such as body composition, diet, and physical activity participation in a larger sample and using a standard glycaemic response to ingesting 50 g of glucose.

### Aim

The aim of the present investigation is to examine the postprandial glucose response of a group of healthy, lean, active young adults without diabetes. Chinese participants who live in China but briefly visited the UK for a three-month summer school at the University were compared to a group of Caucasians matched for age, sex, BMI, body fat percentage, and physical activity. Chinese participants arrived in the UK between 2 and 4 weeks prior to the commencement of the study. To the authors knowledge, this is the first study to assess well-matched young healthy individuals’ glycaemic responses to 50 g glucose and including physical activity, diet, and body-composition data.

## 2. Materials and Methods

After receiving institutional ethical approval, we recruited a sample of 100 Caucasian and Chinese participants, who were healthy (without diabetes) young adults (23.8 (±4.35 years)), lean (body fat ≤ 25%) “healthy” weight (BMI 23.15 ± 2.92 kg/m^2^) through advertisements placed throughout Sheffield Hallam University, Sheffield, UK. Exclusion criteria were diagnosed diabetes, ongoing medication likely to affect glucose metabolism, chronic illness, acute infections, food allergies, smoking, and pregnancy. Following three exclusions due to two participants taking ongoing medication likely to influence glucose metabolism and one participant having high fasting glucose (9.1 mmol/L), 49 Chinese and 48 Caucasian participants met the inclusion criteria.

### 2.1. Procedures

After reviewing the participant information sheet and having an opportunity to ask questions before signing informed consent, all participants completed a food frequency questionnaire (either the Chinese version or the European Prospective Investigation into Cancer and Nutrition (EPIC) version for Caucasians). The Chinese version was translated by two fluent Chinese/English members of the team (SC, LL). Participants completed a 24 h dietary recall and wore a tri-axial (GENEActiv) accelerometer for the day preceding laboratory measurements; participants then attended the research laboratory after a 12 h fast on two separate occasions separated by a week (for the purpose of replication of results). Conditions were standardised for each laboratory session to ensure reliability and validity of the glycaemic responses. Participants were instructed to avoid alcohol and limit intense or unusually high volume physical activity on the day prior to each test day and to eat the same meal at the same time the evening before. Physical activity and diet were recorded in a provided food and physical activity diary. The food and physical activity diaries/accelerometry data were analysed for energy intake, differences in macronutrient intake, and differences in energy expenditure in order to assess whether these were significantly different between tests or between groups.

### 2.2. Anthropometrics

Prior to the physical measurements, participants were asked to void their bowels/bladders. One researcher measured and was shadowed by a second who “confirmed or challenged” the first measurer’s value. Height (without shoes) and body mass (wearing light clothing) were recorded to the nearest 0.1 cm and 0.1 kg, respectively, using scales (SECA 709 mechanical column scales with SECA 220 telescopic measuring rod; SECA Birmingham, United Kingdom). For consistency, participants were asked to wear the same clothes during each visit. Height was recorded at the point of normal breath inspiration with the head orientated in the Frankfort plane. From these measures, BMI was calculated as body mass in kg divided by the height in metres squared and rounded to the nearest 0.1 kg/m^2^. Bioelectrical impedance analysis (BIA) was undertaken on non-conducting foam matting using a BodyStat 1500 (BodyStat Ltd., Douglas, Isle of Man, British Isles). BIA analysis has shown a correlation of 0.83 between bio-electrical impedance and hydrostatic weighing [15]. A constant error in BIA equations suggests an overestimation of body-fat and an underestimation of lean tissue [16]. Measurements were undertaken as per the manufacturer’s instructions following 5 min of supine rest. Percentage body fat and lean weight were recorded to the nearest 0.1% and 0.1 kg, respectively. All measures were taken by one investigator and confirmed by a second.

### 2.3. Blood Glucose and glycated haemoglobin HbA1c

On the first test day, participants provided a capillary blood sample for the determination of HbA1c and baseline blood glucose, using sterilised Softclix lancets (Roche Diabetes Care Ltd., Surrey, UK). Blood glucose was measured via the glucose oxidase method using a Biosen C-Line analyser (EKF Diagnostics, Barleben/Magdeburg, Germany). Whole blood HbA1c was measured on an Alere Afinion AS100 analyser (Livermore, CA, USA). Participants were instructed to consume a 50 g dose of glucose made up to 200 mL with water within 15 min of the baseline glucose test, with all consuming the drink in less than 5 min. Testing glycaemic response using 50 g of glucose has been done in many comparable studies [8,14,15] A timer was started from the first sip of glucose solution, and further measurements were made at 15, 30, 45, 60, 90, and 120 min. Participants returned to the laboratory one week later and the oral glucose test was repeated; the covariance for test–retest reliability was 3.45%. The mean blood glucose responses of the two visits were used for subsequent statistical analysis. Capillary samples of blood were obtained, and each sample was measured in duplicate. An intraclass coefficient of variation of 4.63% was calculated from the Biosen analyser based on 10 replicates. The Biosen has also shown to be valid and reliable elsewhere [17].

### 2.4. Food Frequency Questionnaires (FFQs)

The EPIC FFQ was used with the Caucasian participants and a food frequency questionnaire validated by Shu and colleagues used in the Shanghai Women’s Health Study was used with the Chinese participants [18]. FFQs have been used and validated for collecting data on the average consumption of various food groups. Cade et al. [19] worked on the development and confirmed the validation of the FFQ for use in public health nutrition. Furthermore, FFQs have been defended as essentially accurate in the assessment of number of portions of starchy foods, sugary drinks, rice, and sweets [20].

### 2.5. Physical Activity

Frequency and duration of physical activity was assessed using the short-form 7 day self-report International Physical Activity Questionnaire (IPAQ). Metabolic equivalents (METs) were calculated by combining the self-reported intensity of the exercise with the number of minutes of duration using the physical activity compendium [21]. The IPAQ has acceptable measurement properties—at least as good as other established self-reports for monitoring population levels of physical activity among 18-to-65-year-old adults in diverse settings [21]. In the 24 h prior to attending the laboratory sessions, participants also wore a GENEActiv tri-axial accelerometer (ActivIinsights Ltd. Kimbolton, Cambridgeshire, UK). The accelerometer was attached to the participant’s preferred wrist, and acceleration was sampled at 100 Hz. The technical reliability and validity of this accelerometer has been reported elsewhere [22]. The accelerometer was used to assess the amount of physical activity carried out by participants in the day prior to testing to assess the likelihood of activity affecting the results.

### 2.6. Data Analysis

Incremental area under the curve (iAUC) was calculated using the trapezoidal method outlined by Wolever [23]. Differences between the two ethnic groups for: BMI, age, HbA1c, body fat %, physical activity duration and intensity, baseline glucose, and iAUC were ascertained by independent samples *t*-test after checking for normality. Power calculations were performed on existing data for the primary outcomes of glucose concentration after 1 h and iAUC, showing that 46 participants in each group gave a power of at least 80% (0.80) to detect a 10% difference in 1 h glucose values and iAUC. A repeated measure Analysis of Variation (ANOVA) was performed to analyse any interaction by time and ethnic group. Time to peak, peak glucose, and final glucose concentration (after 2 h) were compared across groups using independent samples *t*-test and 95% CI effect size calculated with Cohen’s *d*. With *d* = ≥ 0.2 ≤ 0.499 a “small” effect, *d* = ≥ 0.5 ≤ 0.799 a “medium” effect, and *d* = ≥ 0.8 a “large” effect [24]. All analyses were conducted using the SPSS Version 24.0 for Windows (IBM, Armonk, NY, USA), and alpha level was set to 0.95.

Ethical approval was granted by the Sheffield Hallam University Food Ethics Research Committee: approval number: SBSREC1314/37.

## 3. Results

### 3.1. Baseline Characteristics

Table 1 highlights the baseline characteristics of the two groups and incudes sex, age, height, body mass, BMI, body fat percentage, and number of MET minutes of physical activity (all *p* > 0.05 for difference). There were significant differences in the intake of several items from the food frequency questionnaire data, with the Chinese eating more rice and the Caucasians eating more starch, sweets, and sugary drinks. Table 2 presents the mean values from the dietary analysis data. Table 3 shows selected items relative to glycaemic response taken form the dietary analysis data and Table 4 present the main outcome measures for the present investigation.

### 3.2. Statistical Analysis

After checking the distribution of data, repeated measures ANOVA with ethnic group as a between-subject factor revealed there was a significant effect of time (*F*(6, 357) = 178.2, *p* < 0.01) and a time × ethnic group interaction (*F*(1, 95) = 3.3, *p* < 0.01). Findings from examining the interactions can be found in Table 4.

Table 2 Chinese/Caucasian FFQ and 24 h recall.

Table 4 shows the main outcome measures: baseline blood glucose, HbA1c, 45 min and 1 h glucose levels, and 2 h iAUC. Baseline values did not differ between the groups 1 h glucose levels and iAUC were both significantly higher in the Chinese group, the analysis for 45 min is included as it is the only time point at which there was a significant difference between groups.

## 4. Discussion

**Figure 1:**
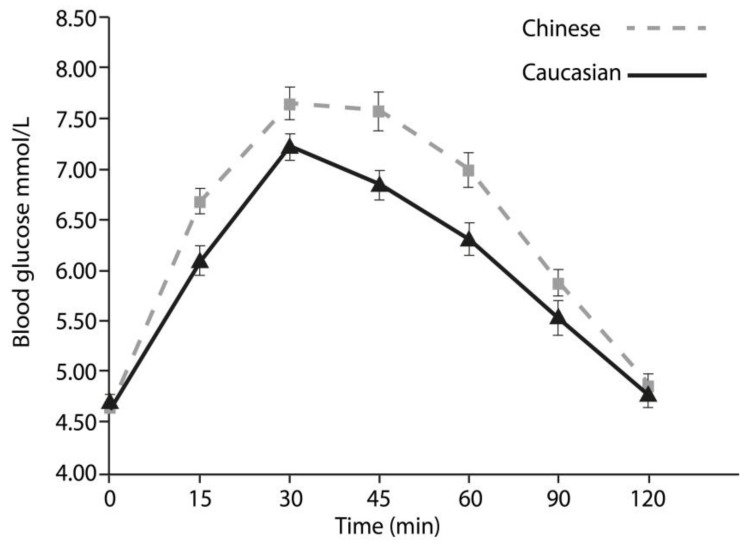
Represents the 2 h iAUC for glucose. The error bars represent standard error. Results are obtained from the average of two laboratory sessions (*p* < 0.001) in mean blood glucose mmol/L values. Chinese (*n* = 49) Caucasian (*n* = 48).

To the authors’ knowledge, this is the first study to examine the 2 h glycaemic response in matched Chinese and Caucasian participants, matched for physical activity, BMI, and body fat percentage. It extends the previous literature which showed similar blood glucose responses, but in participants with significantly different levels of physical activity, BMI, body mass, and no clear indication of body-composition. With these variables accounted for in the present investigation, we discount each of them as explanatory factors in the difference observed between Chinese and Caucasians’ glycaemic responses. These data show clear differences (see Figure 1) in glycaemic response to a standard dose of glucose between two different ethnic groups of healthy, lean, active, young adults, and supports the hypothesis suggesting an ethnic difference, despite non-statistically-significant different values for energy and macronutrient intake, body fat percentage, and physical activity between the two groups.

Previous investigations have shown similar differences in iAUC values [7,8], but between individuals of differing BMI, weight [8], or smaller groups [7]. The present investigation has well-matched groups for BMI, body composition, and physical activity in 49 Chinese and 48 Caucasian individuals measured on two separate occasions. Katoaka et al. [8] speculate that overall rice consumption may be indicated in the higher glycaemic response of Chinese individuals, and in the present investigation it is clear that the pattern of consumption of rice obtained by food frequency data was higher in the Chinese group than it is in the Caucasian group. This work also involved a group of indigenous (students) briefly visiting the UK and their long-term dietary reflections from the food frequency questionnaire represent how they have been eating habitually in China.

The present investigation suggests that some of the most obvious variables in the diet do not appear to relate to the disparity in glycaemic response. It has been proposed that high consumption of white rice is a risk factor for T2D and that populations consuming greater amounts of this staple food stuff have escalated rates of diabetes [25]. In a meta-analysis and systematic review, Hu et al. [25] conclude that white rice consumption increases diabetes risk in a dose-response manner. In the present study, the Chinese participants did eat significantly more rice, yet the intake of high GI foods and starch overall was higher in the Caucasian group. Consumption of a diet high in both GI and GL has been associated with an increased diabetes risk in a study involving 37,846 participants in the Dutch EPIC-NL project [26]. Furthermore, in a randomised crossover design, Wolver and colleagues describe the effects from comparing a low GI diet to that of a high GI diet and suggest both an acute response (29% greater glycaemic rise for high GI breakfasts) and chronic effects on biomarkers (cholesterol and insulin response both negatively indicated in the high GI treatment) [27]. The greater quantity of high GI foods, carbonated beverages, and sweet items consumed by the Caucasians in the present investigation would seem to more than make up for the disparity in the rice consumption of the Chinese group. There could be connections between the type of foods consumed and glycaemic response, namely an increased intake of starch, “sugary foods and candy”, and sugary drinks are obvious factors in elevating blood glucose. However, these appeared to be consumed in greater amounts by the group with the lowest area under the curve (*p* ≤ 0.001 for all). The presence of obvious food related to elevated glycaemic response does not seem to explain our findings. Of course, it is important to note that the quantity of food consumed and what it is combined with (for example white rice alone or sweets alone versus a composite meal with protein added) is likely to affect an individual’s glycaemic response. The only other significant differences, besides the dietary components, occurred in the measures taken for glucose response—specifically the 15, 30, 45 min, and 1 h levels of blood glucose and the overall iAUC. Physical activity levels and body composition were not statistically significantly different, and it appears that other explanatory factors are needed.

The reason for the difference in responses is unknown, but may relate to physiological factors such as different levels of glucose transporter type 4 (Glut-4) [28] and differences in insulin sensitivity between ethnic groups. It may also relate to sudden and extensive urbanisation of the Chinese population [5]. Zhai and colleagues point towards a rapidly shifting pattern of food consumption between 1991 and 2011 (encompassing the time during which the participants in the present investigation were born and growing to maturity), highlighting the fact that this time period has seen a shift toward a declining intake of coarse grains and vegetables, and foods baked, steamed, or boiled at home towards a more Western style of snacking away from home, and with this a clear increase in the intake of snacks high in added sugar. It may be these patterns, or else a combination of physiological and demographic factors which have increased the prevalence and burden of diabetes in China [4,5]. Due to rising diabetes in the Chinese population, further investigation into the effects of diet and any genetic link between diabetes and ethnicity are warranted. Some of the variation in responses by ethnicity have previously been explained by differences in body composition [29] (although not in the Chinese population). This observation did not appear to be the causative factor in the present investigation, however, where body composition between the two groups was not significantly different. Even ignoring the statistical difference and focusing on clinical difference or effect size, the Chinese participants had slightly lower body fat (Cohen’s *d* 0.33).

### Limitations

Food frequency questionnaires were used to assess dietary intake of starch and intake of foods high on the GI scale. Analysis of refined carbohydrate’s effects on blood glucose are affected by what they are consumed with; for example, rice consumed with a source of protein and/or fat will attenuate subsequent rises in blood glucose compared with rice alone, and the data does not allow for this analysis. The cut-off point for Chinese and Caucasian “overweight” BMI values differs (24 kg/m^2^ for overweight in Chinese versus 25 kg/m^2^ in Caucasians). Whilst both the Caucasian and Chinese participants were below this, measuring waist circumference and/or waist to hip ratio would have allowed us to control for central abdominal fat.

## 5. Conclusions

In a well-matched group of healthy, lean, active, young adult Chinese and Caucasians, key lifestyle indicators of glycaemic control (starch and sugar intake) were statistically different between the Chinese and Caucasian participants as well as the glycaemic response. Numerous controlled research projects have now shown an ethnic difference in glycaemic response, including Caucasians, Chinese, and Southeast Indian populations. This study adds to the existing data with two closely matched groups of individuals clearly showing divergent blood glucose responses. Further investigations comparing different ethnic groups matched for body composition and physical activity are warranted.

Further work should identify behaviour related to optimal blood glucose control in Chinese and Caucasian populations and explore the genetic/demographic factors potentially involved in the glycaemic response of Chinese people.

## Figures and Tables

**Table 1:** Participant characteristics.

Variable	Chinese Female	Chinese Male	Caucasian Female	Caucasian Male	*p* *	*d*
Sex, *n* (%)	32 (65)	17 (35)	36 (75)	12 (25)		
Age years (SD)	23.75 (3.08)	22.71 (2.78)	23.81 (3.93)	22.75 (5.16)	0.11	0.01
Height (m)	1.65 (0.07)	1.71 (0.07)	1.67 (0.06)	1.78 (0.08)	0.22	0.26
Body mass (kg)	57.68 (5.61)	73.57 (9.43)	63.09 (6.40)	76.71 (7)	0.08	0.38
BMI (kg/m^2^)	22.46 (3.53)	24.53 (2.95)	22.67 (1.60)	24.42 (3.41)	0.13	0.02
Body fat (%)	23.60 (5.50)	20.38 (6.66)	24.09 (3.54)	24.12 (3.99)	0.31	0.33
Physical Activity (MET min/day)	122.96 (41.20)	113.08 (86.62)	122.34 (41.23)	124.19 (42.46)	0.52	0.04

* *p* values relate to the difference between the Chinese and Caucasian groups assessed by independent sample *t*-test. *d* is the effect size from Cohen’s *d*, also comparing Chinese versus Caucasian groups. BMI: body mass index; MET: metabolic equivalent.

**Table 2:** Dietary assessment within and between groups.

Nutrient/Value	24 h Recall Chinese	FFQ Chinese	*p* for Diff. between 24 h Recall and Chinese FFQ	24 h Recall Caucasian	FFQ Caucasian	*p* for Diff. between 24 h Recall and FFQ Caucasian	*p* for Diff. Chinese/Caucasian FFQ
Energy intake (kcal)	1876 (374)	1987 (275)	0.34	1923 (482)	2001 (376)	0.63	0.78
Carbohydrate%	47 (6.0)	62.4	0.18	42 (7.3)	55 (6.8)	0.81	0.12
Fat%	30 (5.4)	40.12	0.06	34 (3.8)	38.3 (5.11)	0.42	0.84
Protein%	20 (2.3)	22.7 (3.1)	0.41	21.5 (3.4)	23 (2.1)	0.19	0.16
Fibre (grams)	9.9 (3.3)	8.7	0.10	8.7 (2.8)	9.2 (4.0)	0.23	0.72

Table 2 highlights the dietary data recorded from the food frequency questionnaire (FFQ) questionnaires and the 24 h recalls before each laboratory measurement session. The data suggest no significant difference in energy intake, carbohydrate, protein, fat, or fibre, both immediately preceding the experiment and in the participants’ habitual diet.

**Table 3:** Selected items in the FFQ.

	Chinese	Caucasian	*p* for Difference	Cohen’s *d*
* Starchy foods	2.91 (1.69)	5.87 (0.82)	<0.001	1.26
Candy & other sweets	0.31 (0.27)	0.63 (0.78)	0.001	0.9
Sugary drinks	0.80 (0.84)	2.36 (0.54)	<0.001	0.52

Table 3 Showing average daily sugary, starchy food consumption between groups. Values are mean ± (SD) Standard Deviation. *p* value for difference calculated by independent samples *t*-test. Effect size was calculated by Cohen’s *d*. Numbers in brackets represent the standard deviation. * average number of portions intake per day; starchy foods included: noodles, potatoes, bread and pasta.

**Table 4:** Main outcome measures. iAUC: incremental area under the curve.

Variable	Chinese	Caucasian	*p*	*d*
Fasted glucose (mmol/L)	4.7 (0.42)	4.7 (0.50)	0.30	0.10
HbA1c postprandial capillary Blood glucose values	5.01	5.02	0.378	0.70
15 min	6.69 (0.91)	6.11 (1.02)	0.003	0.15
30 min	7.23 (0.92)	7.66 (1.11)	0.041	0.11
45 min	7.59 (1.35)	6.85 (0.97)	<0.001	0.63
60 min	7.00 (1.24)	6.32 (1.13)	<0.001	0.38
90 min	5.54 (1.18)	5.89 (0.89)	0.106	0.08
120 min	4.78 (0.87)	4.85 (0.95)	0.724	0.02
iAUC (120 min)	214.03 (77.49)	156.67 (74.12)	<0.001	0.75

All *p* values relate to an independent samples *t*-test. Effect size was calculated by Cohen’s *d*.

## Trial Registration

The Chinese European study is registered with ClinicalTrials.gov Identifier: NCT02577874.
